# Supplementary material for: Establishment and characterization of new tumor xenografts and cancer cell lines from EBV-positive nasopharyngeal carcinoma
Source: Nat Commun. 2018 Nov 7;9:4663. doi: 10.1038/s41467-018-06889-5 (PMC6220246; doi:10.1038/s41467-018-06889-5)
Supplement: Supplementary file 1 — Supplementary Information [file 41467_2018_6889_MOESM1_ESM.pdf]

Supplementary Information

Establishment and characterization of new tumor xenografts and cancer cell lines from EBV-positive nasopharyngeal carcinoma

Lin *et al*.

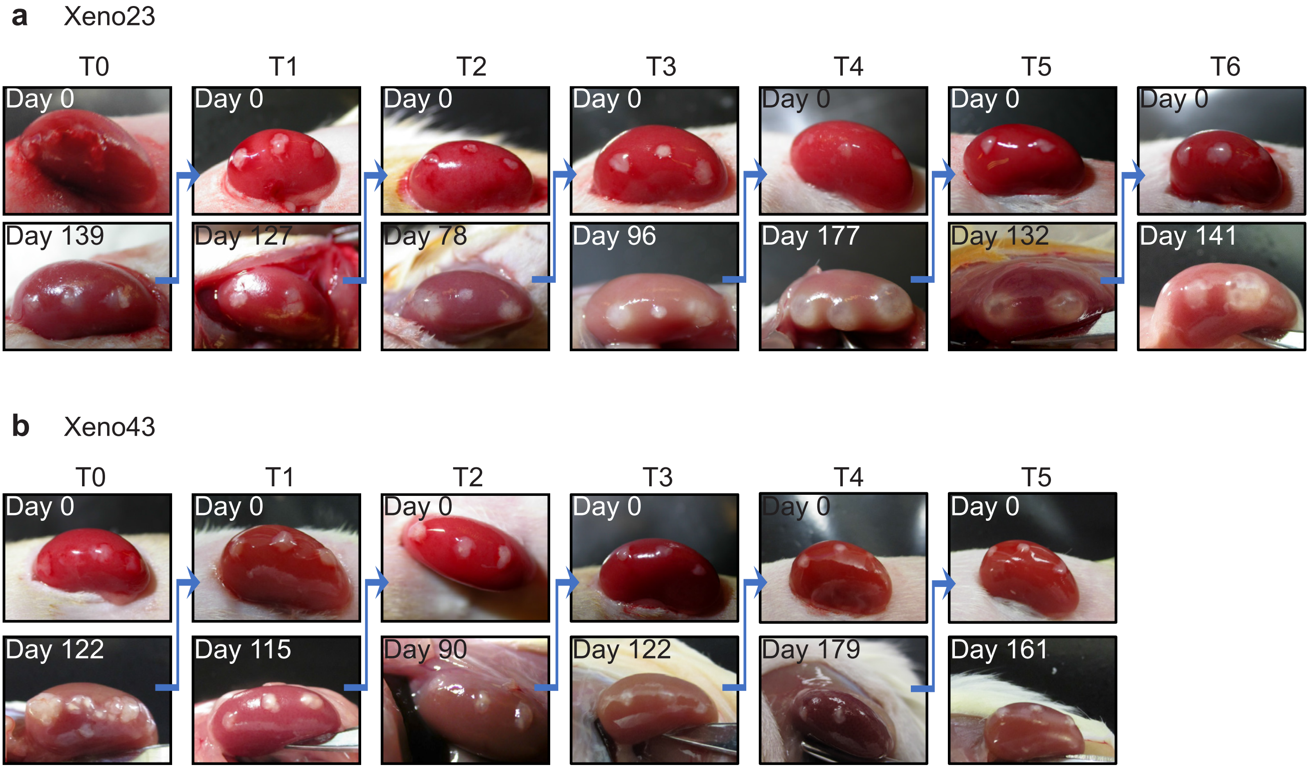

Supplementary Figure 1. Serial transplantations of NPC PDXs.

(a) Serial transplantation of NPC tissues was performed to maintain Xeno23 in subrenal growth in NOD/SCID mice. From T0 (before transfer) to T3 (the 3rd transfer), the growth of Xeno23 was very slow. Robust growth was observed from T4 (the 4th transfer) onwards.

(b) The growth of Xeno43 was observed at T0 after implanting NPC tumor tissues under renal capsule of NOD/SCID mice and keeping for growth for 122 days. However, the size of this PDX shrank in mice during T1 (the 1st transfer) to T5 (the 5th transfer), and eventually the growth stopped.

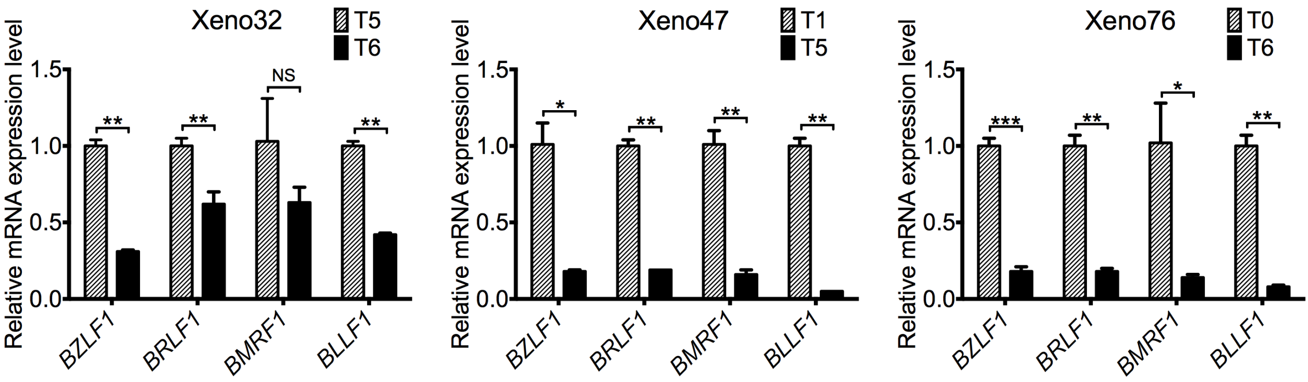

Supplementary Figure 2. Expression of EBV lytic genes in NPC PDXs.
Expression of lytic EBV genes including *BZLF1*, *BRLF1*, *BMRF1* and *BLLF1*, in three newly established PDXs (Xeno32, 47 and 76) was quantified by real-time PCR. The expression levels between early passages and late passages were compared. Higher expression levels of EBV lytic genes were revealed in PDXs at early passages. Data are shown as mean ± SD from 3 independent experiments. * $p < 0.05$; ** $p < 0.005$; *** $p < 0.0005$; NS: not significant, in a two-tailed t-test.

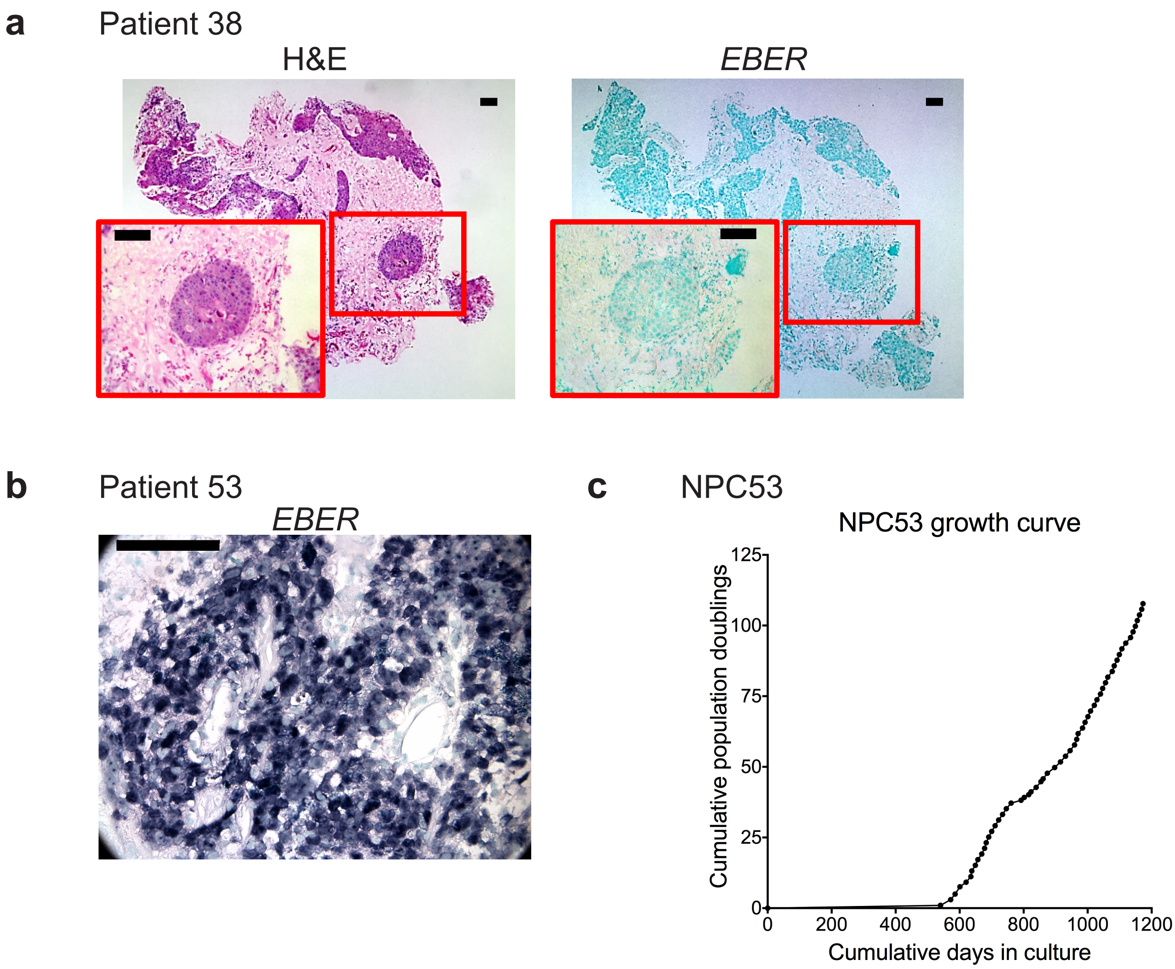

Supplementary Figure 3. Establishment of two EBV-ve NPC cell lines.
(a) Histological examination of NPC tumor from patient 38. Left panel: H&E staining showing the presence of carcinoma cells. Right panel: *EBER* ISH shows that the carcinoma cells in NPC tumor tissue of patient 38 were EBV-ve. Scale bars, 100 µm.
(b) *EBER* ISH of NPC tumor from patient 53. The presence of EBV infection in the original patient tissue of NPC53 was confirmed. Scale bar, 100 µm.
(c) Growth curve of NPC53 cell line. For the establishment of this cell line, slow growth of NPC53 was observed regarding outgrowth from explanted tumor tissues. It took more than 500 days for the outgrowing epithelial cells to become confluency in culture flask. Accelerated population doublings were revealed in the later passages of NPC53.

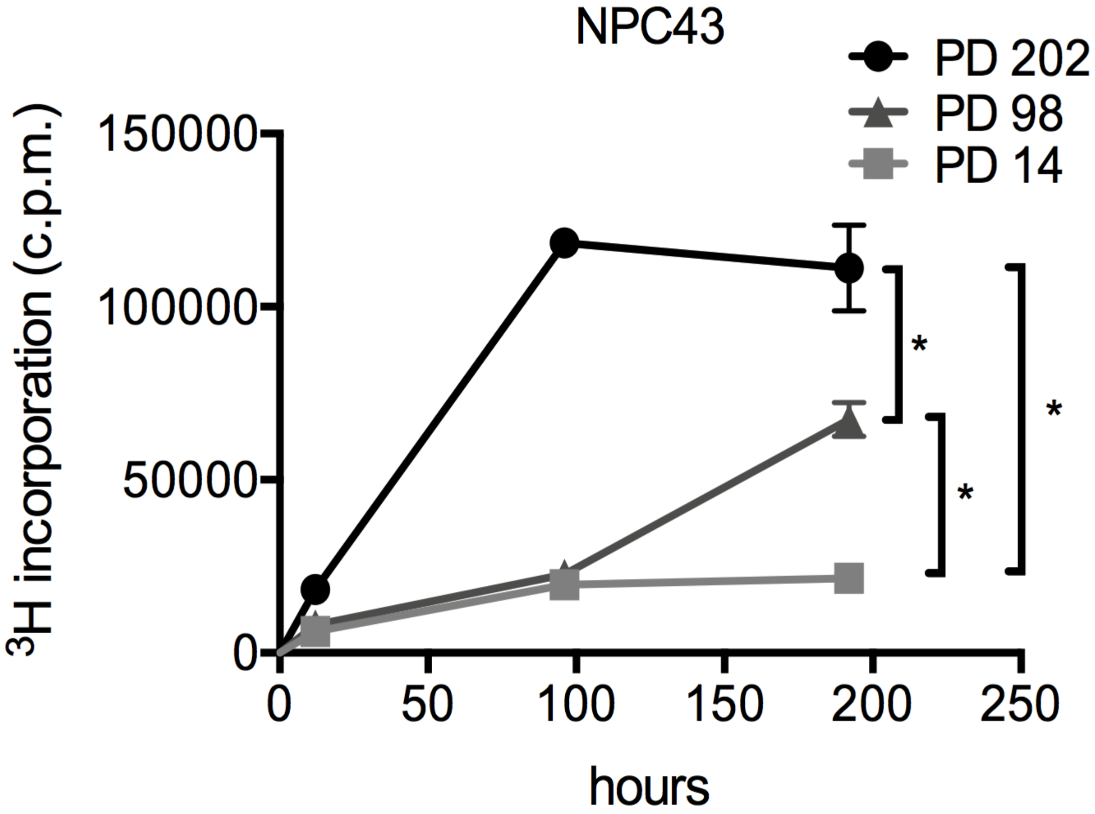

Supplementary Figure 4. Comparison of cell proliferation rates of NPC43 at different passages.

The proliferation rate of NPC43 cells was evaluated by thymidine incorporation assay. Results revealed the increased thymidine incorporation in NPC43 at higher population doublings. Data are shown as mean ± SD from 3 independent experiments. * $p < 0.05$ in a two-tailed *t*-test.

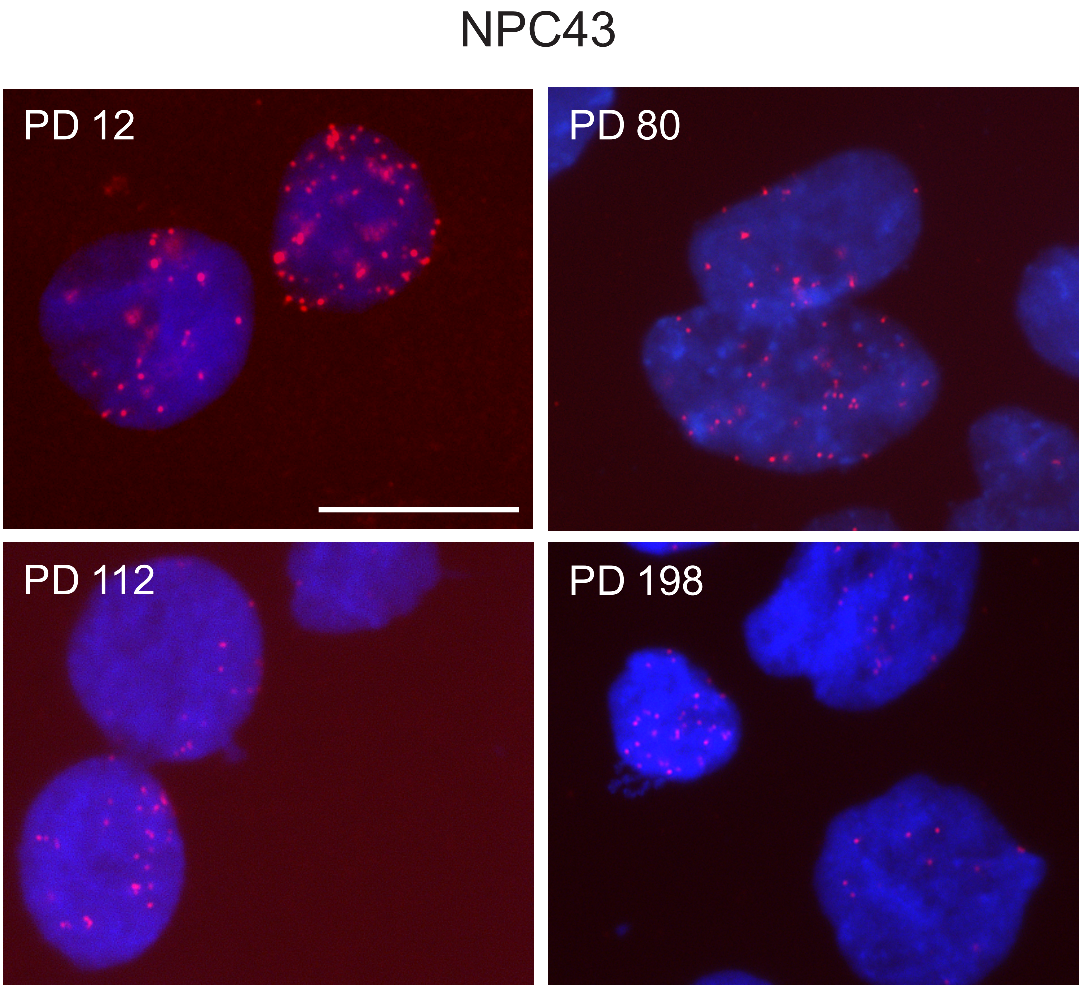

Supplementary Figure 5. Determination of the presence of EBV episomes in NPC43 by FISH analysis.

Representative images showing the presence of EBV genomes in NPC43 at PD 12, 80, 112 and 198, respectively. A gradual decrease in the number of EBV genomes per cell was observed in NPC43 at late passages. Scale bar, 10 μm.

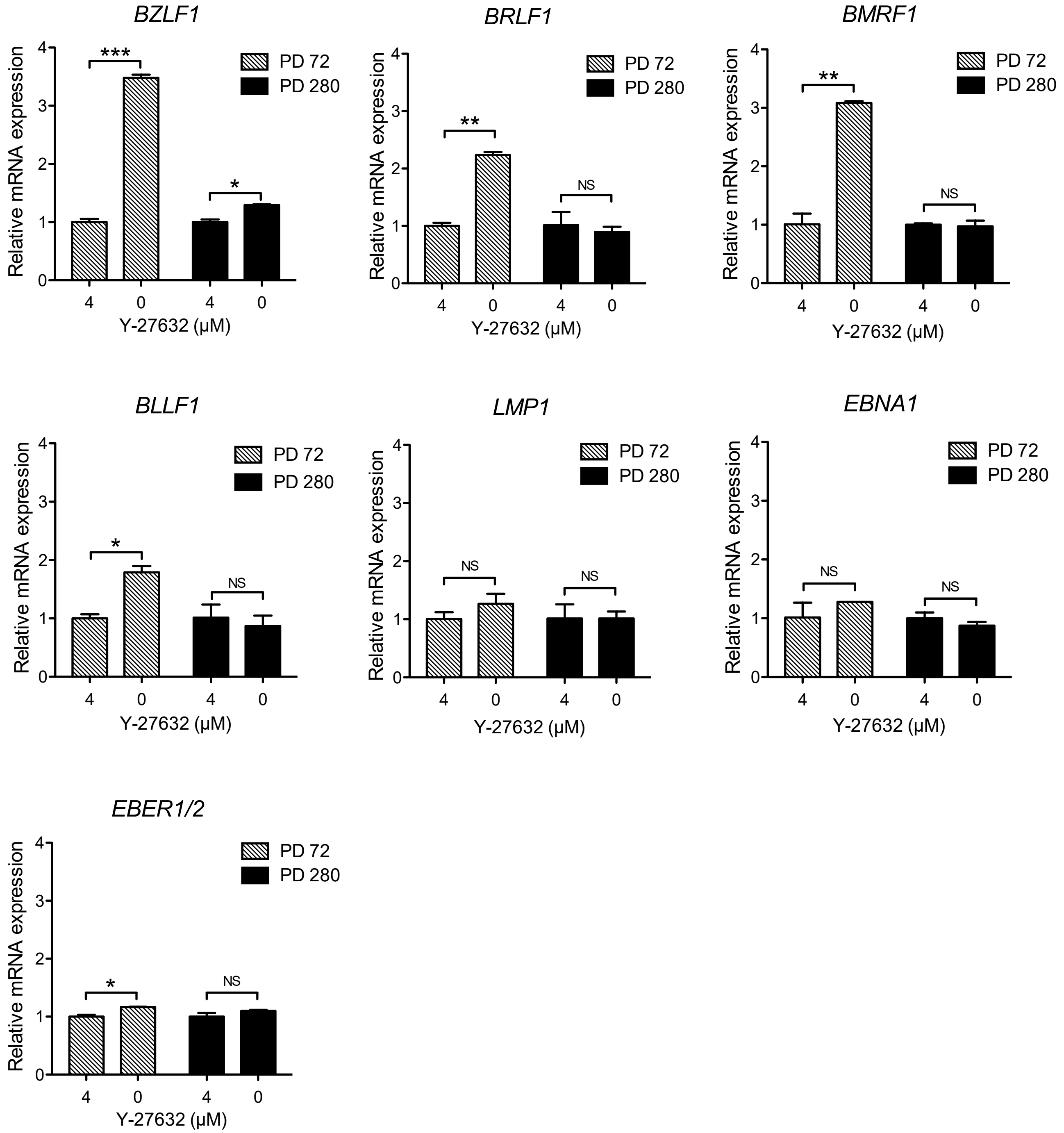

Supplementary Figure 6. Expression of EBV lytic and latent genes in NPC43 at early and late passages upon removal of Y-27632.

The changes of EBV gene expression upon removal of Y-27632 were evaluated in NPC43 cells at PD 72 (early passage) and PD 280 (late passage) by real-time PCR. Briefly, Y-27632 was removed from the culture medium for 48 hours before cells were harvested for analysis. Increased expressions of lytic EBV genes (*BZLF1*, *BRLF1*, *BMRF1* and *BLLF1*) were observed in NPC43 at early passage, but not at later passage upon deprivation of Y-27632. Meanwhile, no significant changes of expression levels of latent EBV genes (*EBNA1*, *LMP1* and *EBER*) upon removal of Y-27632 were found. Data are shown as mean ± SD from 3 independent experiments. * $p < 0.05$; ** $p < 0.005$; *** $p < 0.0005$; NS, not significant in a two-tailed *t*-test.

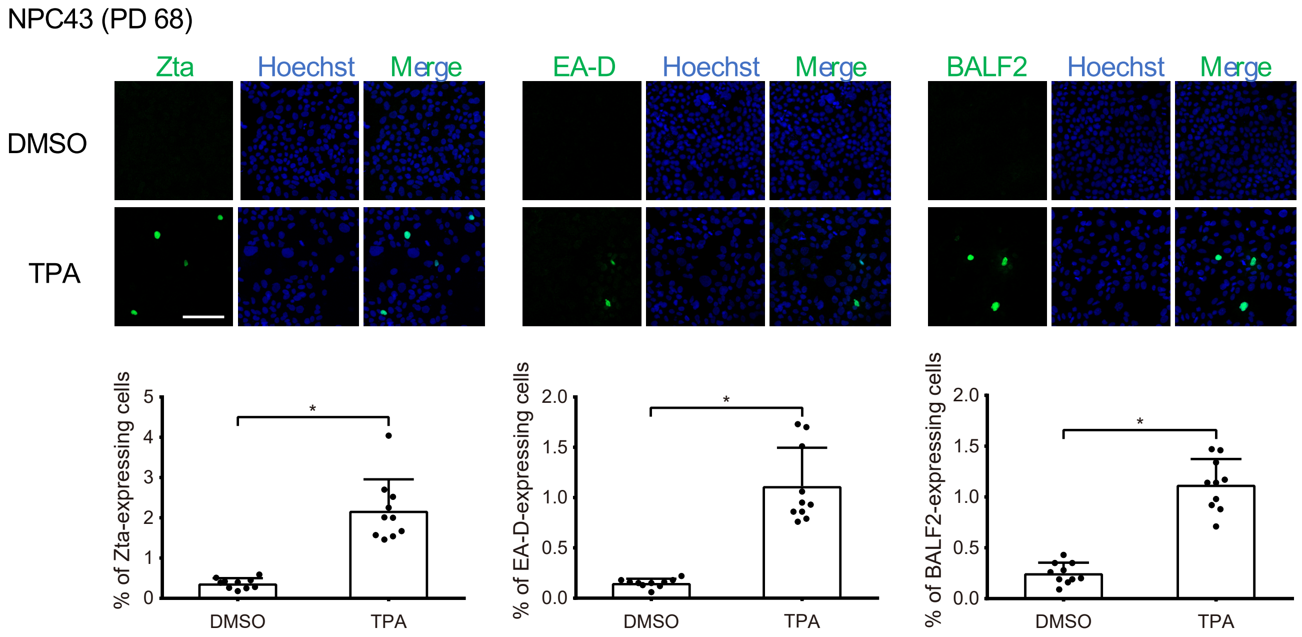

Supplementary Figure 7. Determination of EBV lytic protein expressions by immunofluorescent staining in NPC43 upon the treatment with TPA.

Expression of lytic EBV proteins, including Zta, EA-D and BALF2, were examined in NPC43 cells at PD 68 with or without the treatment with TPA for 48 hours. Top panel: representative images showing the staining of Zta, EA-D and BALF2 in NPC43 cells; Bottom panel: quantification of the percentage of positive-stained cells (including Zta, EA-D and BALF2) in NPC43 was performed by cell number counting. Data are shown as mean ± SD from 10 different microscopic views with over 2,000 cells included. * $p < 0.05$ in a two-tailed Z-test. Scale bar, 100 μm.

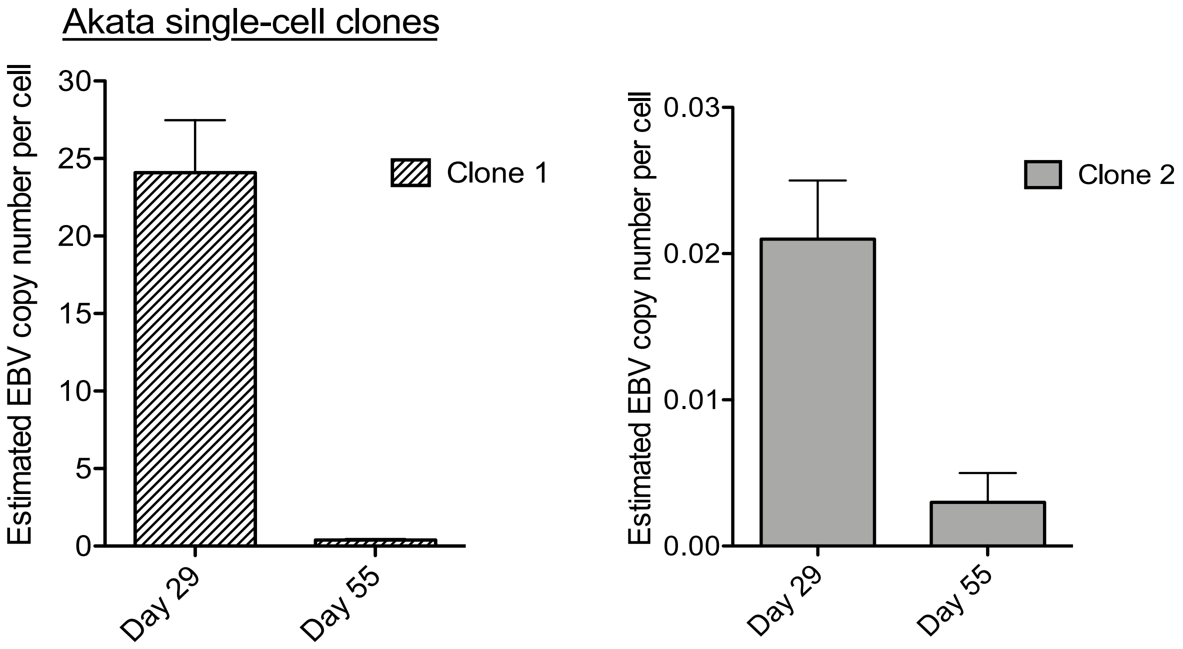

Supplementary Figure 8. Evaluation of EBV copy number in Akata single-cell clones with NPC43-EBV infection.

NPC43-EBV was collected from the supernatant of NPC43 cells after lytic induction as stated. EBV-ve Akata cells were infected by NPC43-EBV by co-culture method, and subsequently subjected for single-cell sorting to generate single-cell clones. Two representative Akata clones were collected for EBV copy number determination 29 and 55 days after cell sorting. A significant decrease was observed in the average EBV copy number in Akata single-cell clones infected with NPC43-EBV after prolonged culture. Data are shown as mean ± SD from 3 independent experiments.

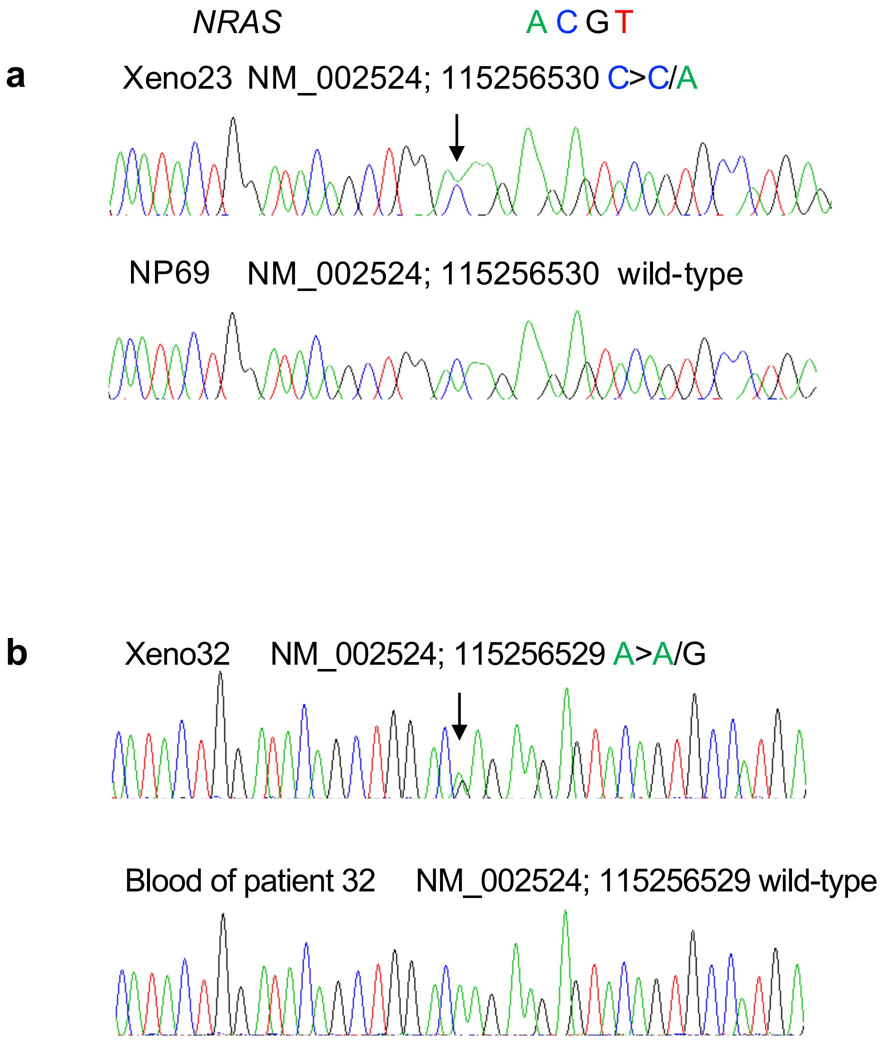

Supplementary Figure 9. Validation of somatic mutations in *NRAS* by Sanger sequencing.
(a) Arrow indicates a heterozygous SNV as C>C/A verified in Xeno23 at the coordinate of Chr11: 115256530. The sequence of the same region in NP69 cells was illustrated as wild-type control.
(b) Arrow indicates a heterozygous SNV as A>A/G verified in Xeno32 at the coordinate of Chr11: 115256529. The sequence of the same region in the blood of patient 32 serves as wild-type control.

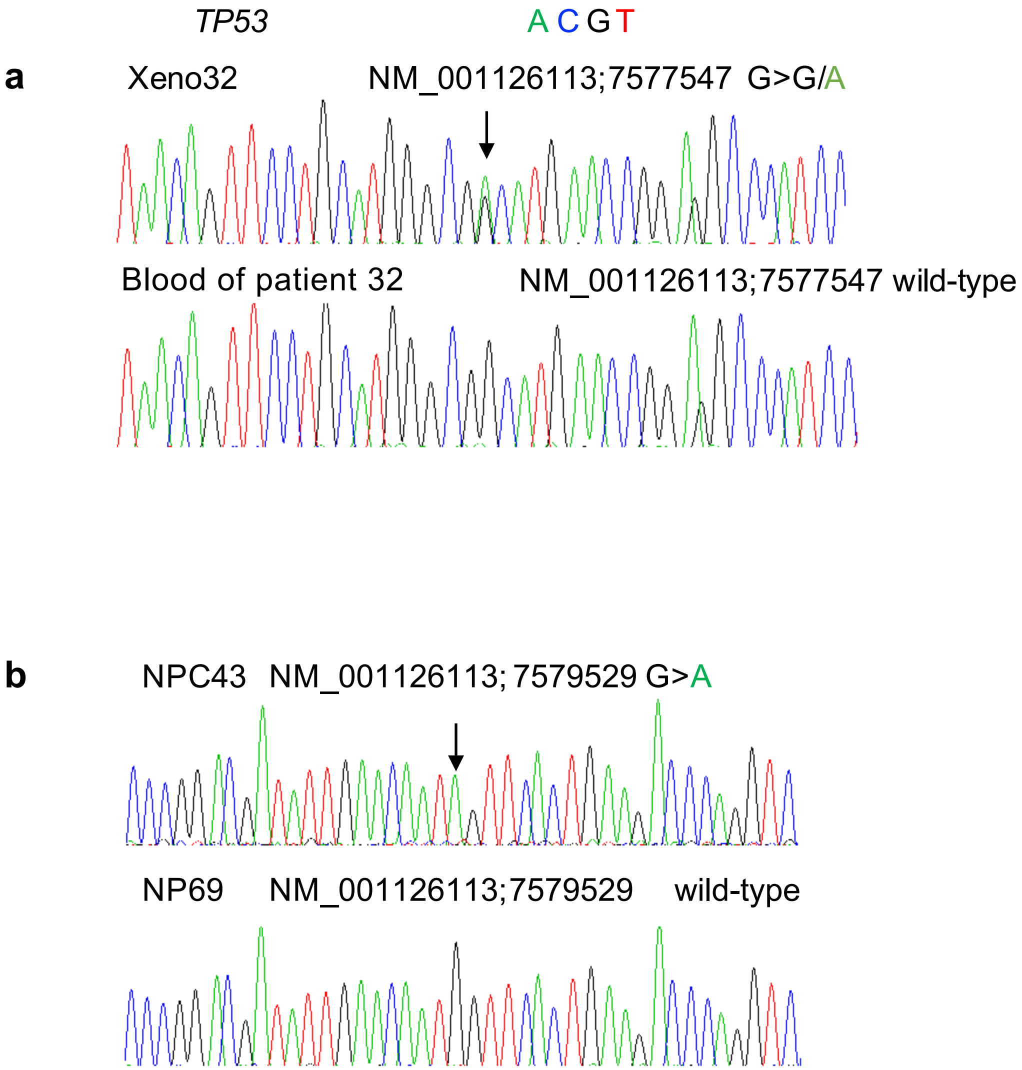

Supplementary Figure 10. Validation of somatic mutations in *TP53* by Sanger sequencing.
(a) Arrow indicates a heterozygous missense SNV as G>G/A verified in Xeno32 at the coordinate of Chr17: 7577547. The sequence of the same region in the blood of patient 32 serves as wild-type control.
(b) Arrow indicates a homozygous nonsense SNV as G>A verified in NPC43 at the coordinate of Chr17: 7579529. The sequence of the same region in NP69 cells was illustrated as wild-type control.

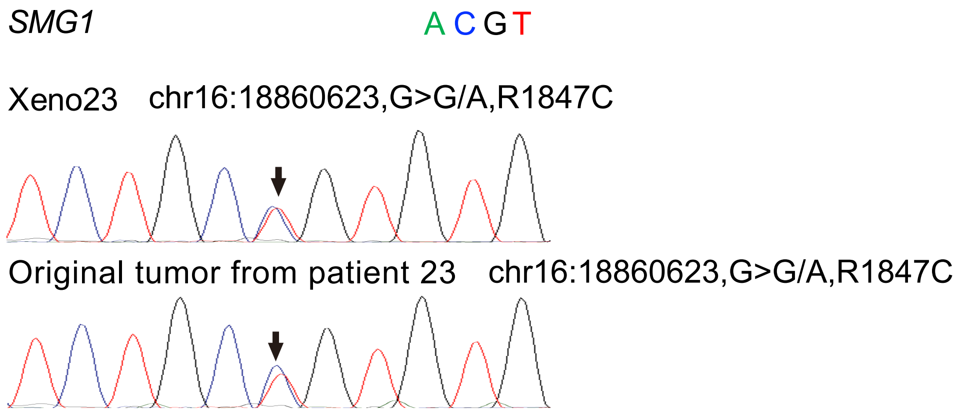

Supplementary Figure 11. Validation of an *SMG1* SNV by Sanger sequencing.
Arrows indicate a heterozygous missense mutation as G>G/A verified in Xeno23 and its corresponding patient tumor tissue at the coordinate of Chr16: 18860623.

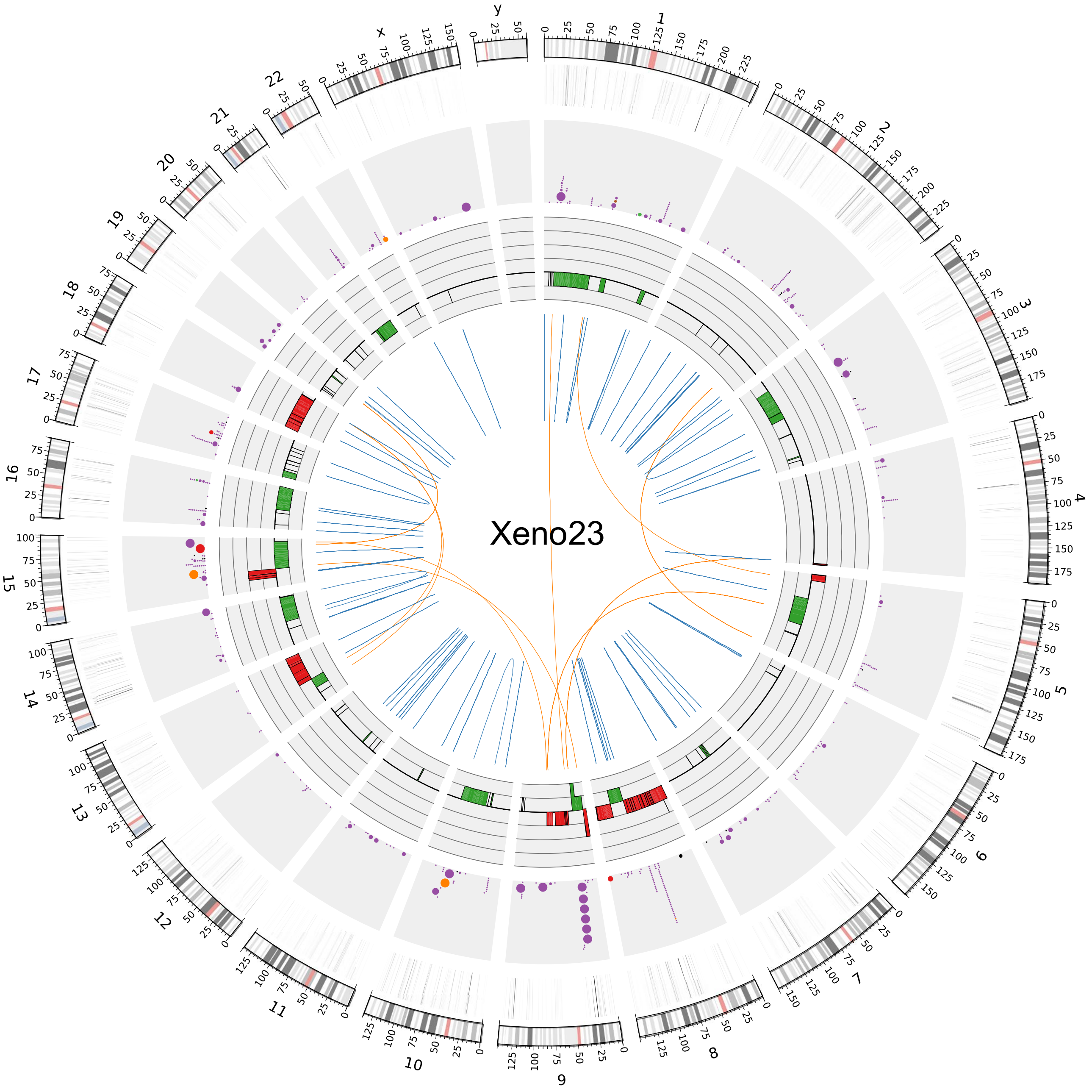

Supplementary Figure 12. Circos plot depicting the somatic alterations in Xeno23.
The following information is illustrated in order from inner to outer rings based on WGS data analysis: Ring 1 (innermost) for structural variants (orange, inter-chromosomal rearrangement; blue, intra-chromosomal rearrangement); Ring 2 for copy number variants (green, copy number loss; red, copy number gain; range: -2 to +4); Ring 3 for non-synonymous single-nucleotide variants or small indels (purple, missense; black, splicing; orange, nonsense; red, frameshift; green, inframe indel; brown, others) with allele frequency indicated by the size of each dot (0%, 20%, 40%, 60%, 80%, 100% or more); Ring 4 for density of SNVs/small indels (0 per Mbps, 20, 40, 60, 80, 100, 120, 140, 160 or more); Ring 5 (outermost) for chromosome scale at 1 Mbps (shades of gray, cytobands; red, centromere).

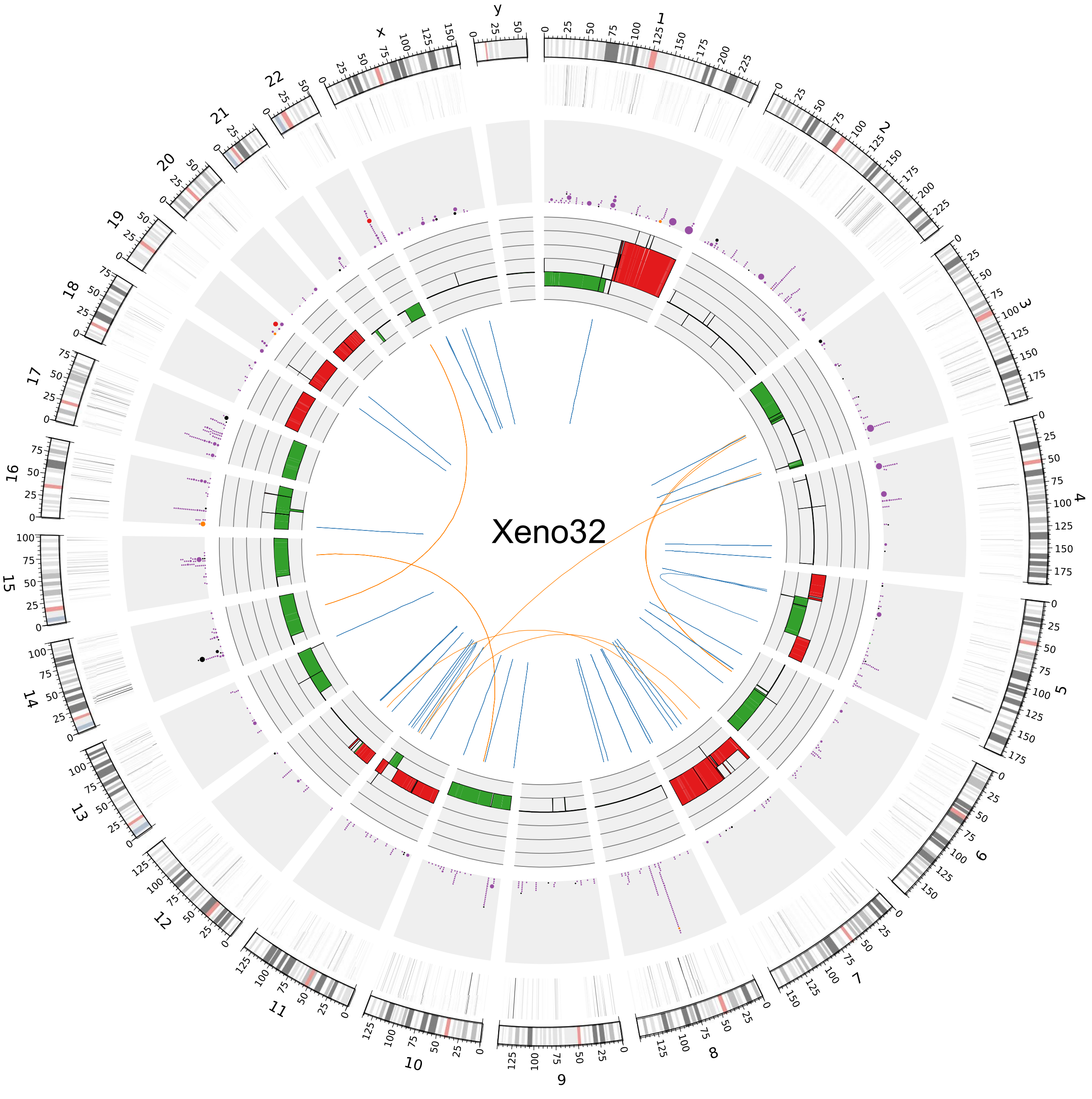

Supplementary Figure 13. Circos plot depicting the somatic alterations in Xeno32.
The following information is illustrated in order from inner to outer rings based on WGS data analysis: Ring 1 (innermost) for structural variants (orange, inter-chromosomal rearrangement; blue, intra-chromosomal rearrangement); Ring 2 for copy number variants (green, copy number loss; red, copy number gain; range: -2 to +4); Ring 3 for non-synonymous single-nucleotide variants or small indels (purple, missense; black, splicing; orange, nonsense; red, frameshift; green, inframe indel; brown, others) with allele frequency indicated by the size of each dot (0%, 20%, 40%, 60%, 80%, 100% or more); Ring 4 for density of SNVs/small indels (0 per Mbps, 20, 40, 60, 80, 100, 120, 140, 160 or more); Ring 5 (outermost) for chromosome scale at 1 Mbps (shades of gray, cytobands; red, centromere).

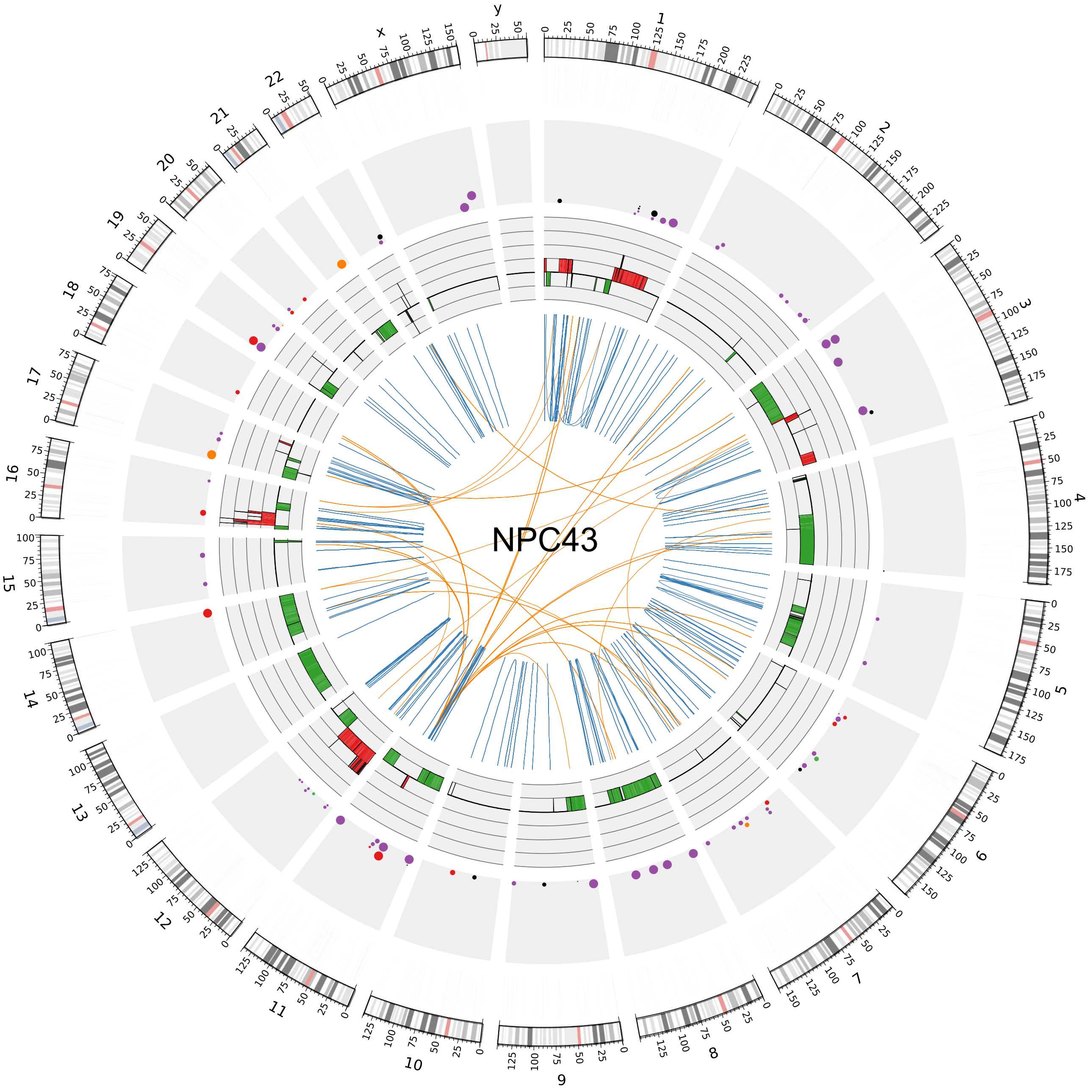

Supplementary Figure 14. Circos plot depicting the somatic alterations in NPC43.
The following information is illustrated in order from inner to outer rings based on WGS data analysis: Ring 1 (innermost) for structural variants (orange, inter-chromosomal rearrangement; blue, intra-chromosomal rearrangement); Ring 2 for copy number variants (green, copy number loss; red, copy number gain; range: -2 to +4); Ring 3 for non-synonymous single-nucleotide variants or small indels (purple, missense; black, splicing; orange, nonsense; red, frameshift; green, inframe indel; brown, others) with allele frequency indicated by the size of each dot (0%, 20%, 40%, 60%, 80%, 100% or more); Ring 4 for density of SNVs/small indels (0 per Mbps, 20, 40, 60, 80, 100, 120, 140, 160 or more); Ring 5 (outermost) for chromosome scale at 1 Mbps (shades of gray, cytobands; red, centromere).

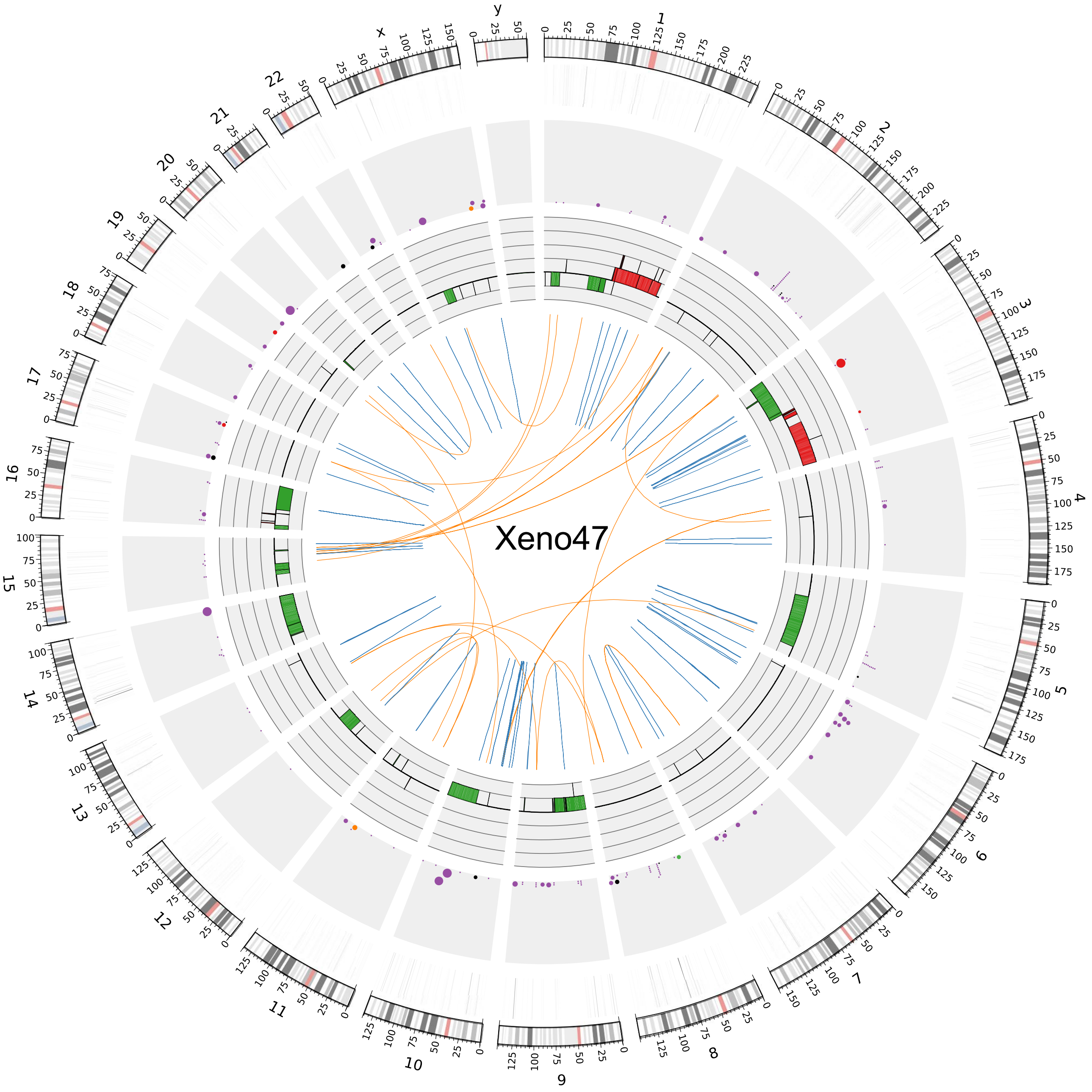

Supplementary Figure 15. Circos plot depicting the somatic alterations in Xeno47.
The following information is illustrated in order from inner to outer rings based on WGS data analysis: Ring 1 (innermost) for structural variants (orange, inter-chromosomal rearrangement; blue, intra-chromosomal rearrangement); Ring 2 for copy number variants (green, copy number loss; red, copy number gain; range: -2 to +4); Ring 3 for non-synonymous single-nucleotide variants or small indels (purple, missense; black, splicing; orange, nonsense; red, frameshift; green, inframe indel; brown, others) with allele frequency indicated by the size of each dot (0%, 20%, 40%, 60%, 80%, 100% or more); Ring 4 for density of SNVs/small indels (0 per Mbps, 20, 40, 60, 80, 100, 120, 140, 160 or more); Ring 5 (outermost) for chromosome scale at 1 Mbps (shades of gray, cytobands; red, centromere).

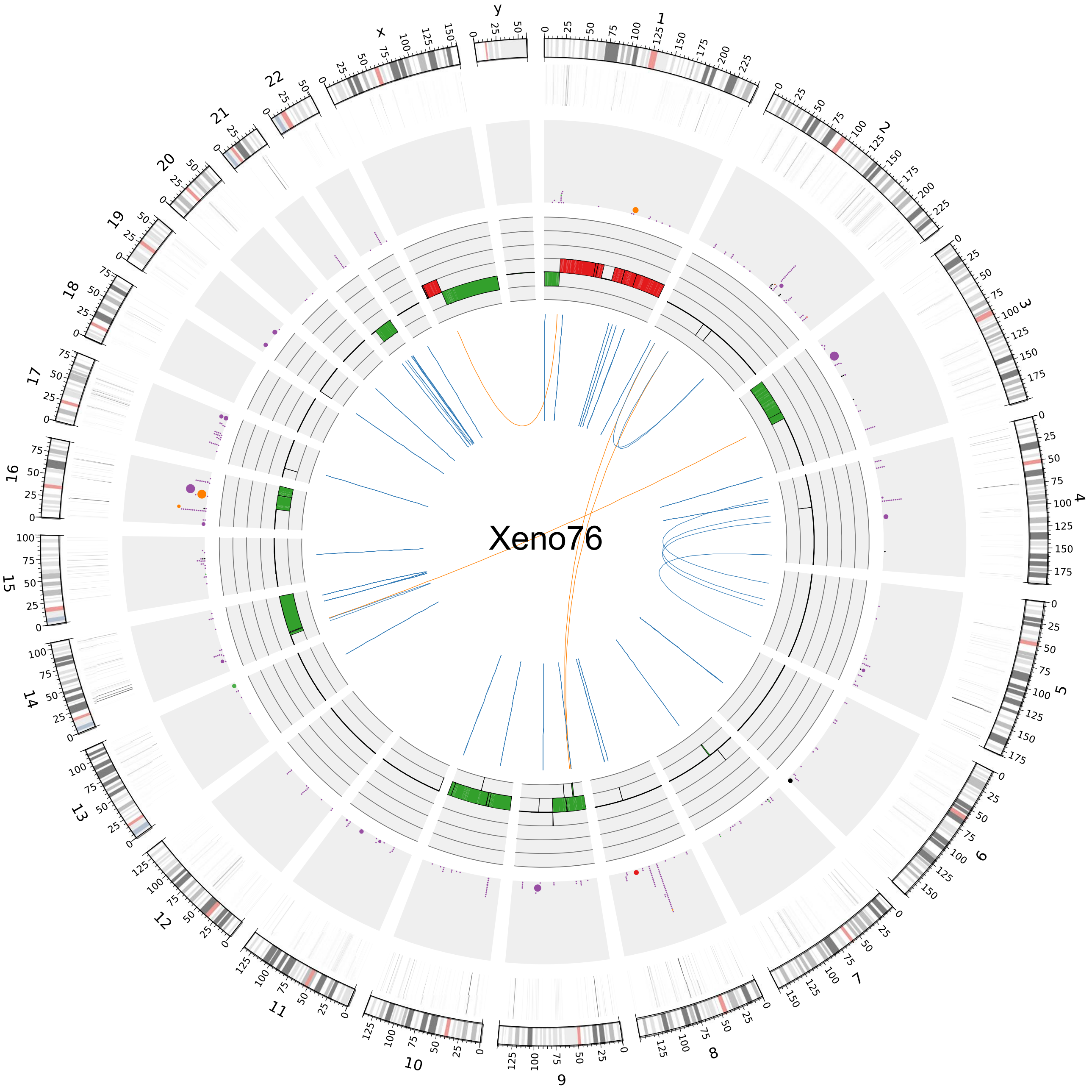

Supplementary Figure 16. Circos plot depicting the somatic alterations in Xeno76.
The following information is illustrated in order from inner to outer rings based on WGS data analysis: Ring 1 (innermost) for structural variants (orange, inter-chromosomal rearrangement; blue, intra-chromosomal rearrangement); Ring 2 for copy number variants (green, copy number loss; red, copy number gain; range: -2 to +4); Ring 3 for non-synonymous single-nucleotide variants or small indels (purple, missense; black, splicing; orange, nonsense; red, frameshift; green, inframe indel; brown, others) with allele frequency indicated by the size of each dot (0%, 20%, 40%, 60%, 80%, 100% or more); Ring 4 for density of SNVs/small indels (0 per Mbps, 20, 40, 60, 80, 100, 120, 140, 160 or more); Ring 5 (outermost) for chromosome scale at 1 Mbps (shades of gray, cytobands; red, centromere).

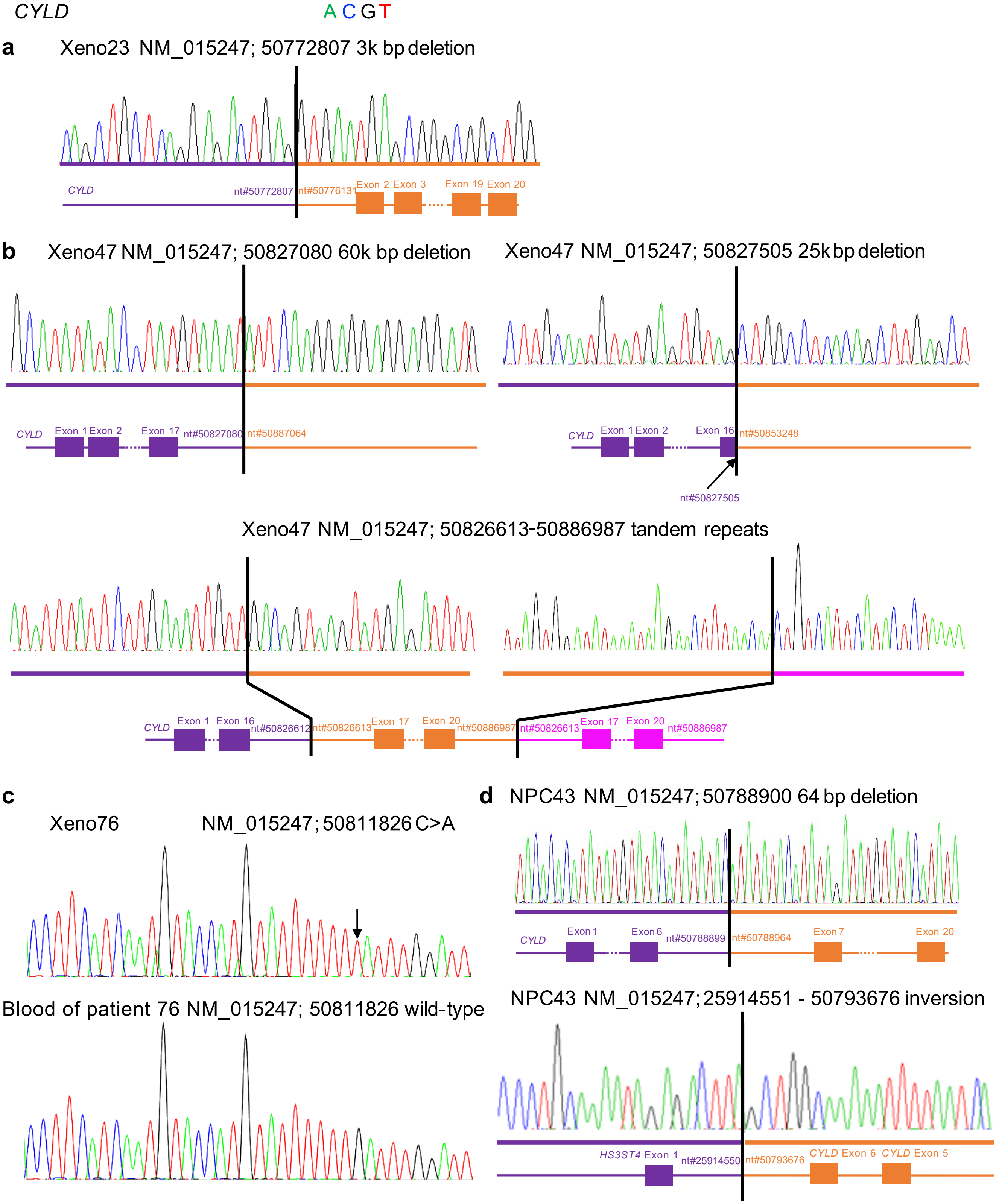

Supplementary Figure 17. Validation of somatic mutations in CYLD by Sanger sequencing.
(a) A deletion of over 3 kb in length in Chr16 (50772807 to 50776131) was verified in Xeno23.
(b) Deletions of 60 kb (50827080 to 50887064) and 25 kb (50827505 to 50853248), and tandem repeats of 50826613 to 50886987 in Chr16 were verified in Xeno47.
(c) Arrow indicates a homozygous missense mutation as C>A verified in Xeno76 at the coordinate of Chr16: 50811826. The sequence of the same region in the blood DNA of patient 76 was included as wild-type control.
(d) A 64 bp deletion (50788900 to 50788863) and an inversion (25914551 to 50793676) in Chr16 were verified in NPC43.

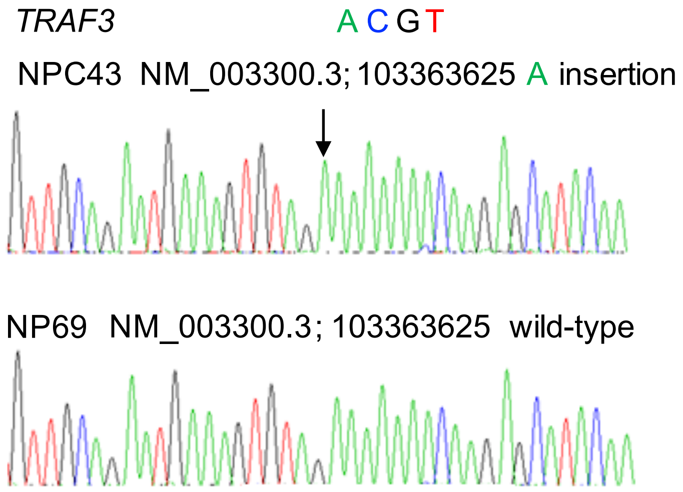

Supplementary Figure 18. Validation of *TRAF3* mutation by Sanger sequencing.
Arrow indicates a homozygous adenine (A) insertion verified in NPC43 at the coordinate of Chr14: 103363625. The sequence of the same region in NP69 cells was included as wild-type control.

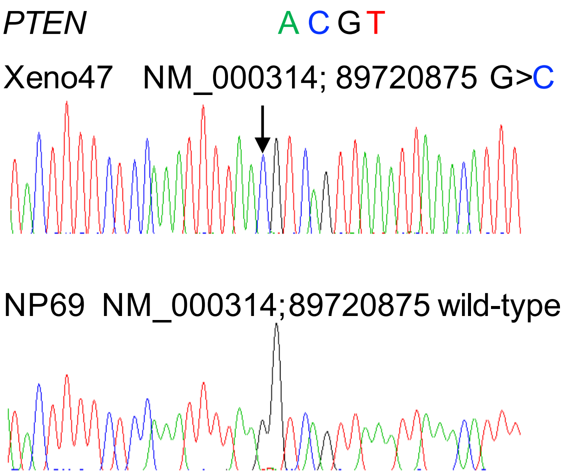

Supplementary Figure 19. Validation of *PTEN* missense mutation by Sanger sequencing. Arrow indicates a homozygous missense mutation as G>C verified in Xeno47 at the coordinate of Chr10: 89720875. The sequence of the same region in NP69 cells was included as wild-type control.

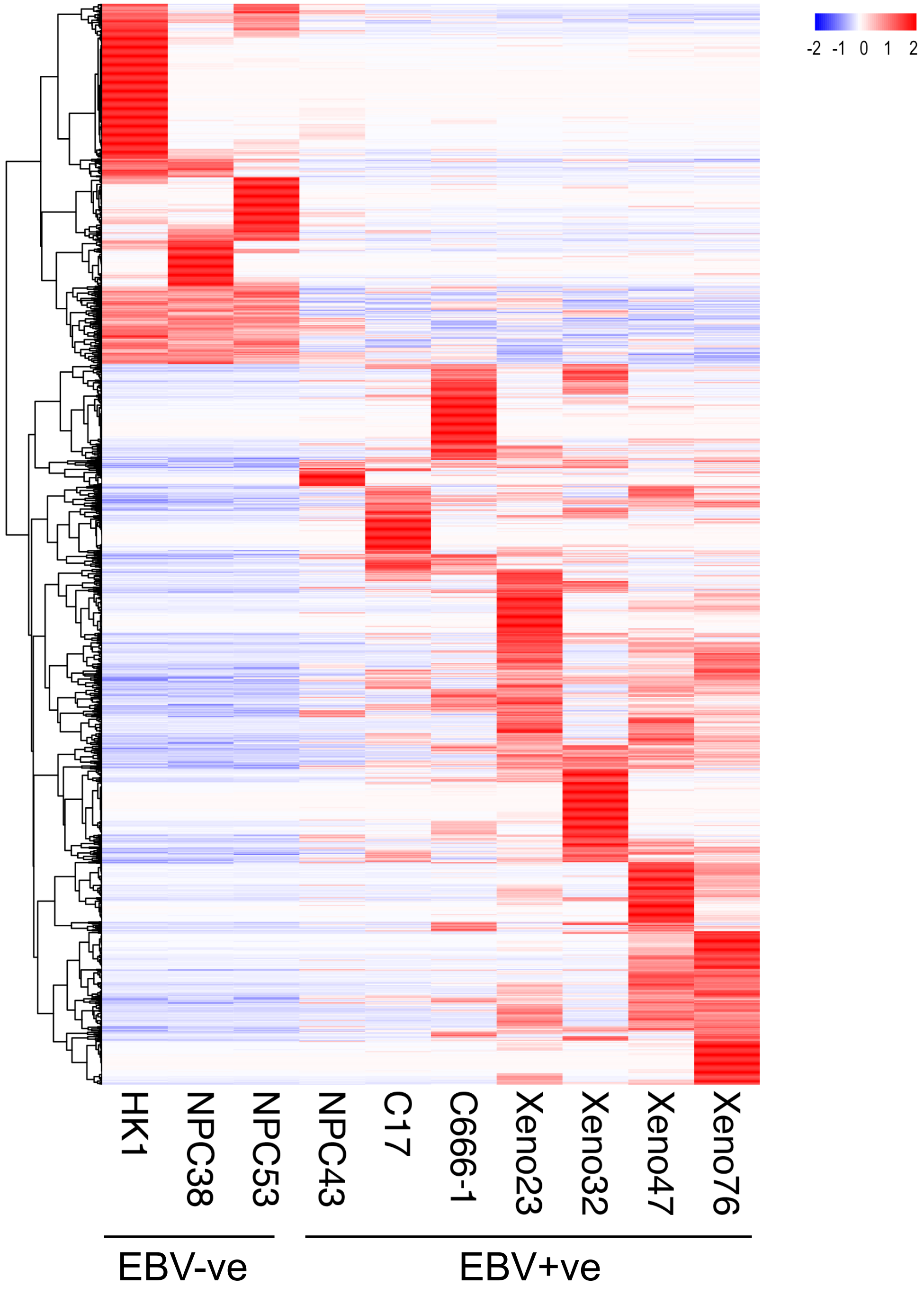

Supplementary Figure 20. Differentially expressed genes in EBV-ve and EBV+ve NPC cell lines and xenografts.

Heatmap showing 1974 differentially expressed genes in EBV-ve (HK1, NPC38 and NPC53) and EBV+ve (NPC43, C17, C666-1, Xeno23, 32, 47 and 76) samples. Briefly, RNA sequencing was performed to characterize the transcriptome profiles of newly established NPC PDXs and cell lines, together with well-established NPC cell lines, including HK1 (EBV-ve, derived from well differentiated squamous carcinoma), C17 (EBV+ve, recently developed from C17 xenograft) and C666-1 (EBV+ve, derived from xenograft X666). Gene expression levels as normalized read counts quantified by RSEM were subjected to EBSeq comparison to determine the differentially expressed genes between EBV+ve and EBV-ve cohorts. The genes with FDR q-value below 0.05 were considered as significantly upregulated or downregulated. Heatmap was drawn and gene pattern clustering was analyzed using pheatmap R package. Red indicates high relative expression while blue indicates low relative expression. Range: -2 to 2.

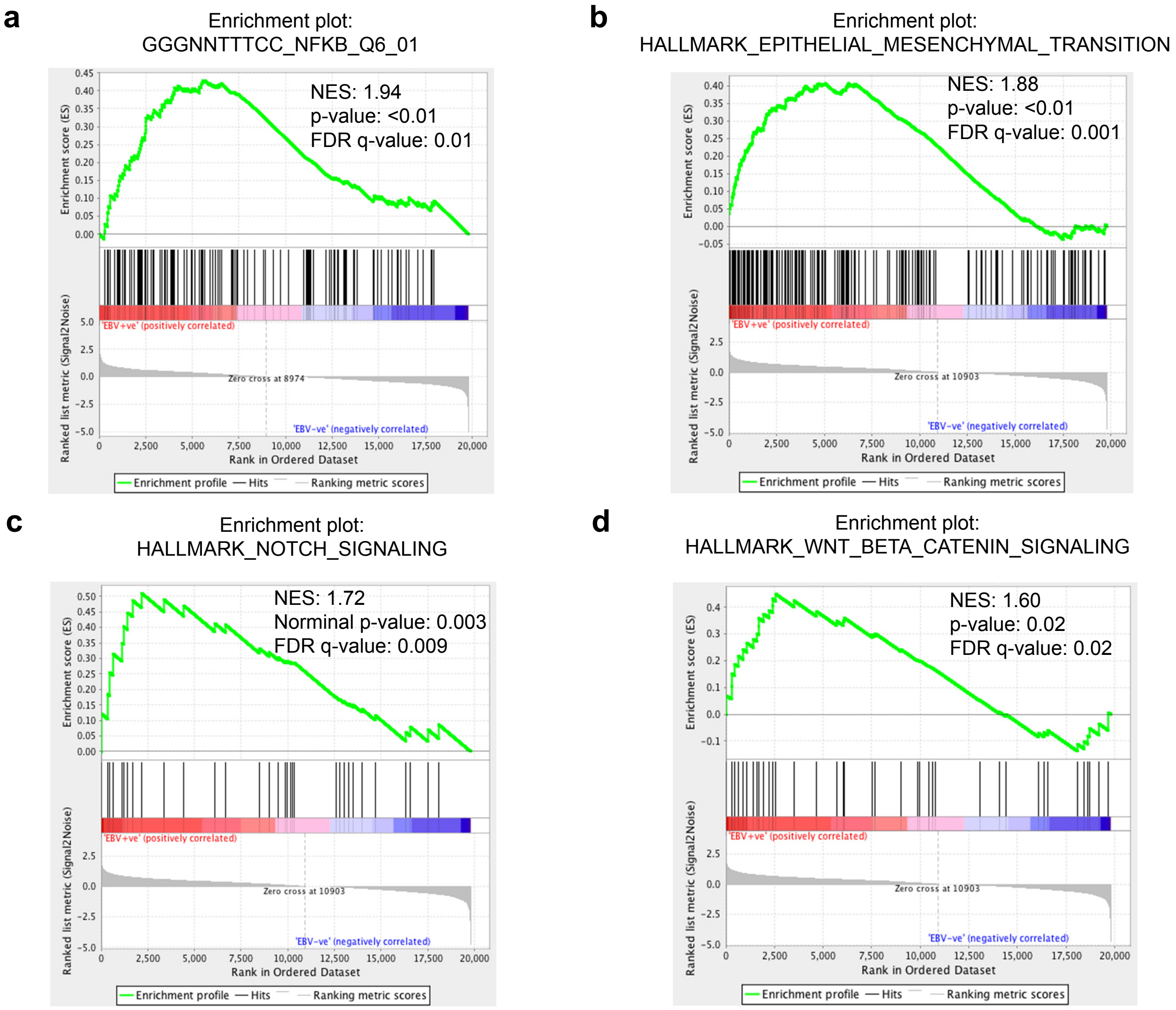

Supplementary Figure 21. Activation of NF-κB and other cancer-associated pathways in EBV+ve NPC cells and xenografts.

RNA sequencing was performed and gene expression levels were quantified as stated in Materials and Methods. The expression levels of all protein-coding genes were subject to gene set enrichment analysis (GSEA) for pathway analysis. The expression of genes in two cohorts was compared, including EBV-ve (HK1, NPC38 and NPC53) and EBV+ve (C666-1, C17, NPC43, Xeno23, 32, 47 and 76). For a brief illustration for GSEA enrichment plot, enrichment score (ES) indicates the degree to which the specified gene set is overrepresented at the top or bottom of a ranked list of total protein-coding genes in all PDXs/cell lines. The normalized enrichment score (NES) computes the density of modified genes in the dataset with the random expectancies, normalized by the number of genes found in each gene cluster. The false discovery rate (FDR) is calculated by comparing the actual data with 1000 Monte-Carlo simulations. For each enrichment plot, the top portion reveals the running ES for the specified gene set; the middle portion indicates where the member of the gene set appears in the ranked list of genes; the bottom portion shows the value of the ranking metric as a measurement of a gene's correlation with a phenotype (EBV+ve or EBV-ve). A significant enrichment in GGGNNTTTCC_NFKB gene set (a) was indicated by GSEA results, which was a gene set previously identified by NF-κB regulatory motif analysis in promoter regions of human genome. EBV+ve samples exhibited enhanced activation of NF-κB pathway evidenced by the upregulated NF-κB targets, as compared to the EBV-ve cohort. GSEA results also revealed significant enrichment in HALLMARK Epithelial-Mesenchymal Transition gene set (b), HALLMARK Notch signaling gene set (c), and HALLMARK Wnt-beta-catenin signaling gene set (d) with gene upregulation in EBV+ve cohort as compared to the EBV-ve counterpart, with NES, p-value and FDR q-value indicated.

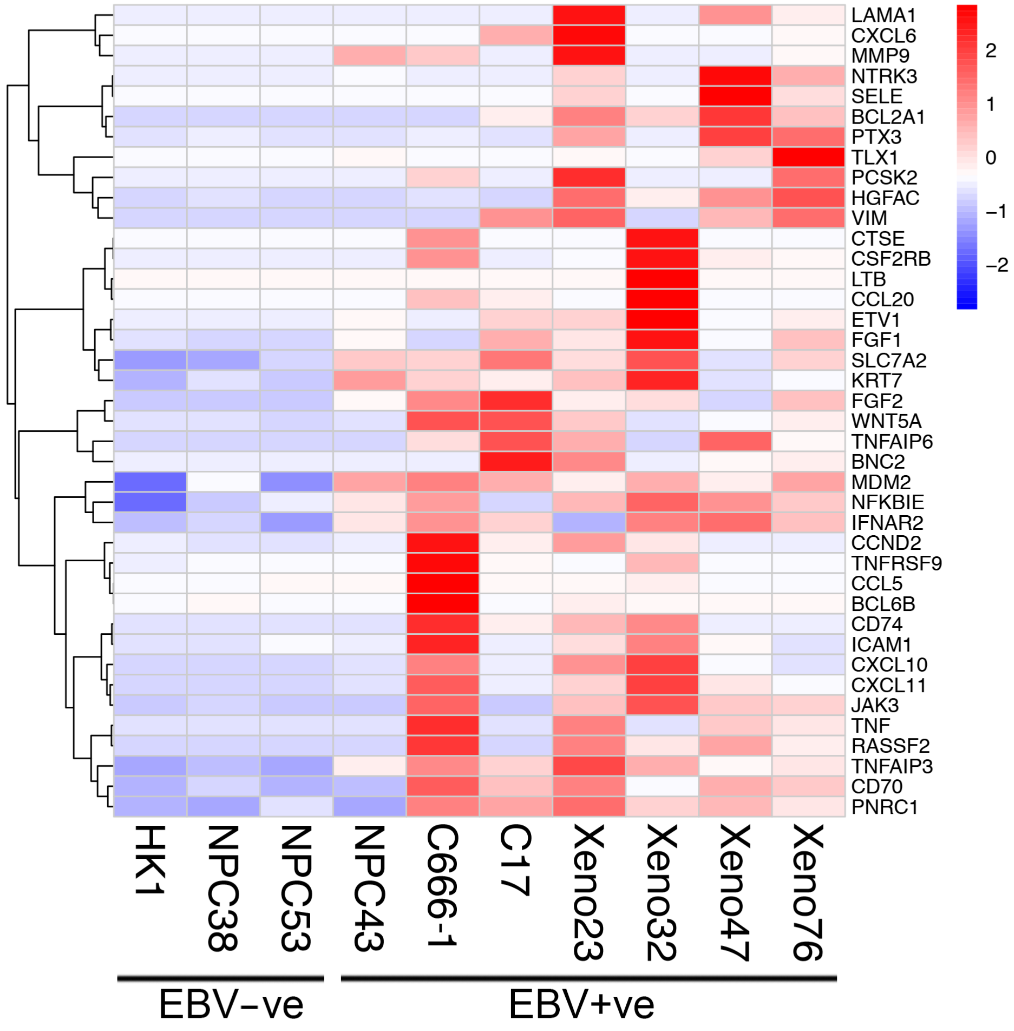

Supplementary Figure 22. Significant upregulated expression of NF-κB targets in EBV+ve NPC cells and xenografts revealed by RNA sequencing analysis.

Heatmap illustrating the upregulated NF-κB targets in EBV+ve cohort (including NPC43, C666-1, C17, Xeno23, 32, 47 and 76) as compared to EBV-ve cohort (including HK1, NPC38 and NPC53). RNA sequencing was performed and gene expression levels were quantified as stated. Putative NF-κB targets identified by previous reports (illustrated in Materials and Methods) were selected for comparison by EBSeq between EBV+ve and EBV-ve cohorts. The genes with FDR q-value below 0.05 were considered as significantly upregulated NF-κB targets in EBV+ve cohort. Heatmap was drawn and gene pattern clustering was analyzed using pheatmap R package. Red indicates high relative expression while blue indicates low relative expression. Range: -2 to 2. The upregulated expression pattern of NF-κB targets is consistent with the genomic profiles of EBV+ve NPC models with commonly identified loss-of-function mutations in negative regulators of NF-κB pathway.

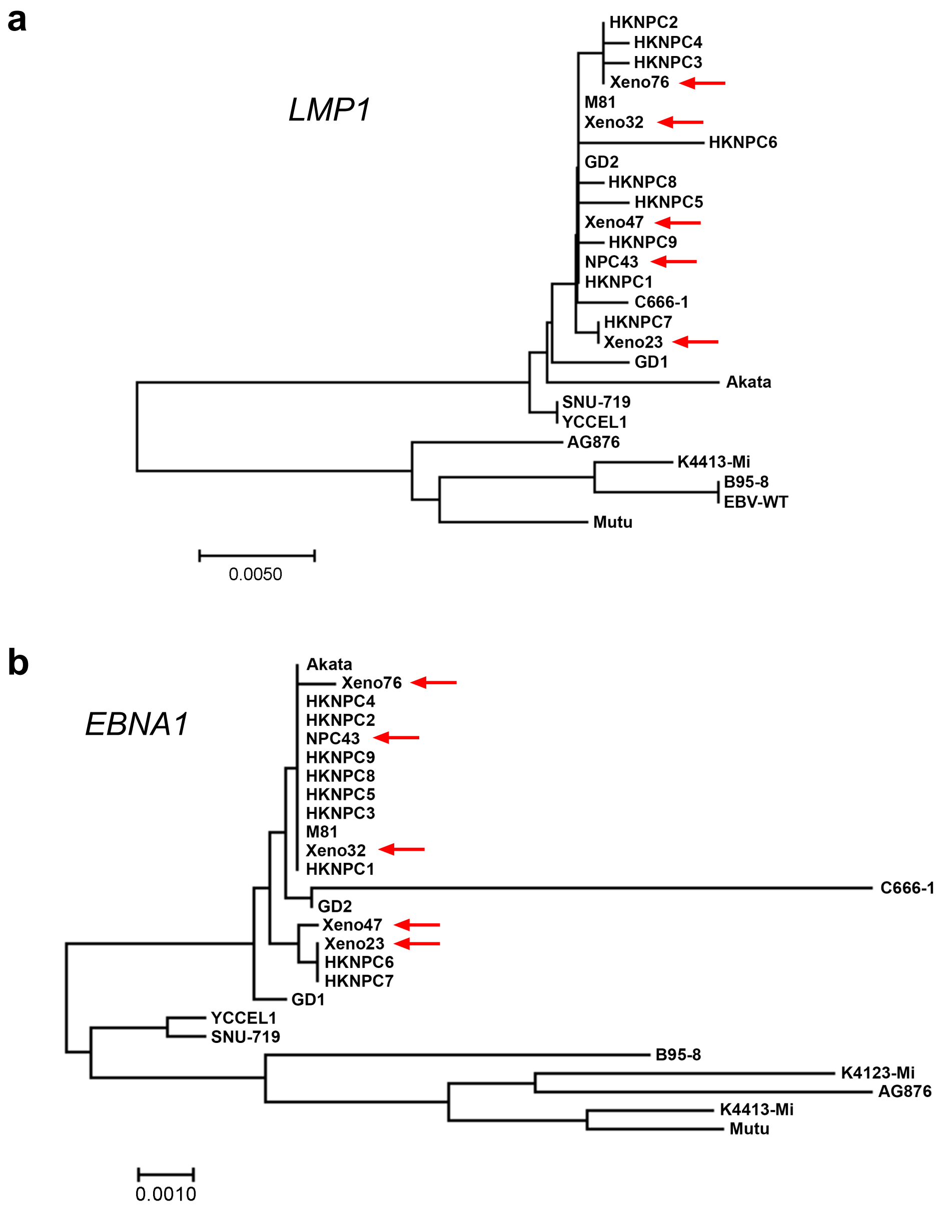

Supplementary Figure 23. Phylogenetic analysis of EBV genes.

Phylogeny trees depicting the phylogenetic similarity between the sequences of protein coding regions of *LMP1* gene (a) and *EBNA1* gene (b) in the newly assembled EBV genomes (Xeno23, 32, 47, 76 and NPC43) and other publicly available sequences of EBV. Briefly, the non-human and non-mouse reads from WGS were aligned to the reference EBV genome (NC_007605) using BWA software. The generated BAM files were subjected to SAMtools software for pile-up files and assessment of coverage of reads. After reads trimming and calculation of average coverage of reads, high-quality reads were assembled using Velvet with k-mer length optimized. The sequences of EBV genes in the new assembled EBV sequences were subjected to the multiple sequence alignment with MAFFT with other publicly available sequences. The aligned sequences were visualized and edited using Jalview software. Phylogenetic analysis was performed using MEGA7 by neighbour-joining algorithm, and phylogenetic tree was generated and drawn to scale, with branch lengths in the same units as those of the evolutionary distances used to infer the phylogenetic tree. The evolutionary distances were computed using the Maximum Composite Likelihood method. The results indicate that the *LMP1* and *EBNA1* gene sequences in Xeno23, 32, 47, 76 and NPC43 show close phylogenetic similarity with those in NPC-EBV, including HKNPC1-9, M81, GD2 and C666-1, but are distinct from the gene sequences in other EBV genomes, including EBV from gastric cancer (YCCEL1 and SNU719) and EBV from B lymphoma (B95.8 and Mutu).

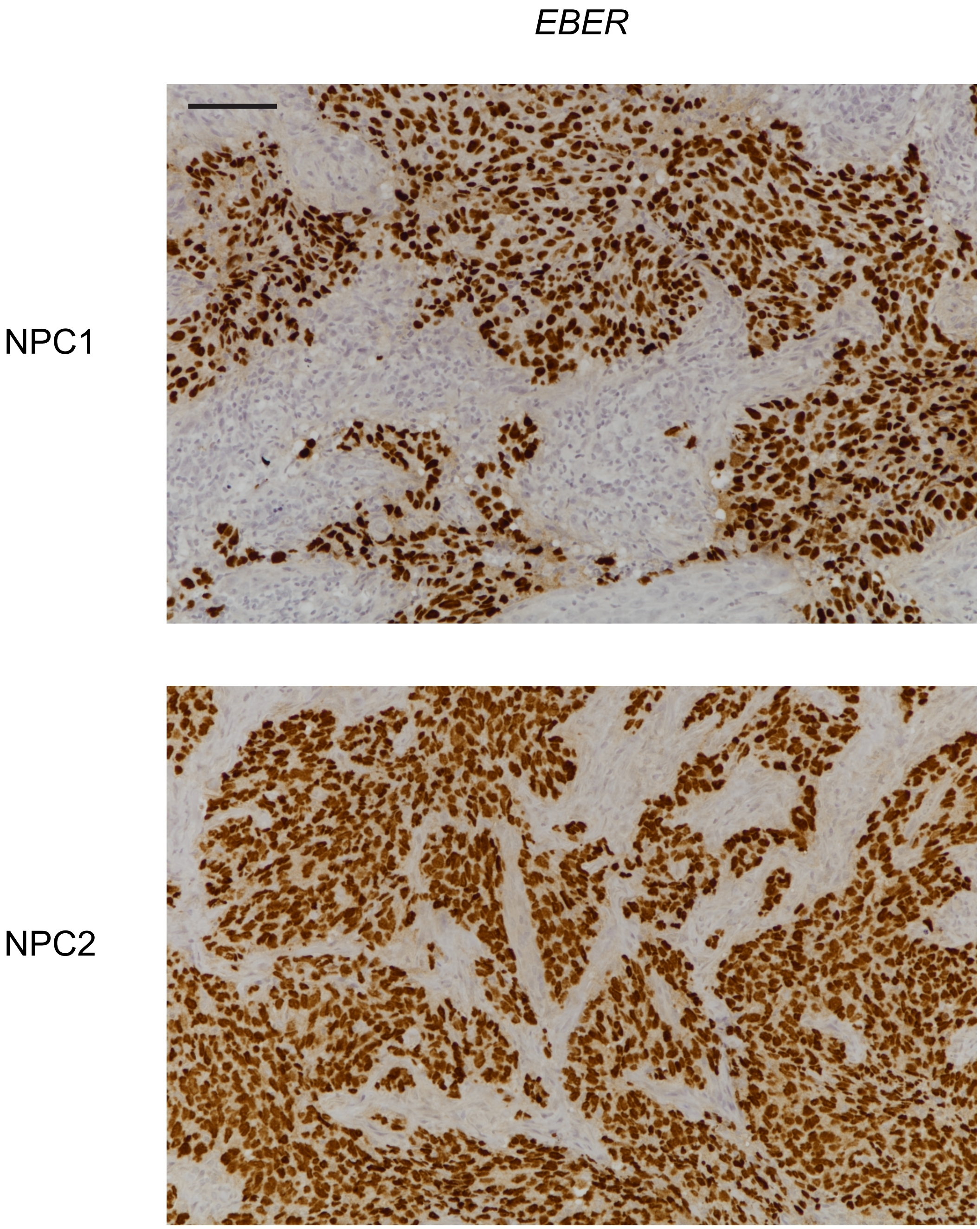

Supplementary Figure 24. EBER staining of NPC1 and NPC2 clinical specimens. *EBER* staining was performed using RNAscope® specific probe. Positive EBV infection status in these two clinical specimens was confirmed by the expression of *EBER*. Scale bar, 100 μm.

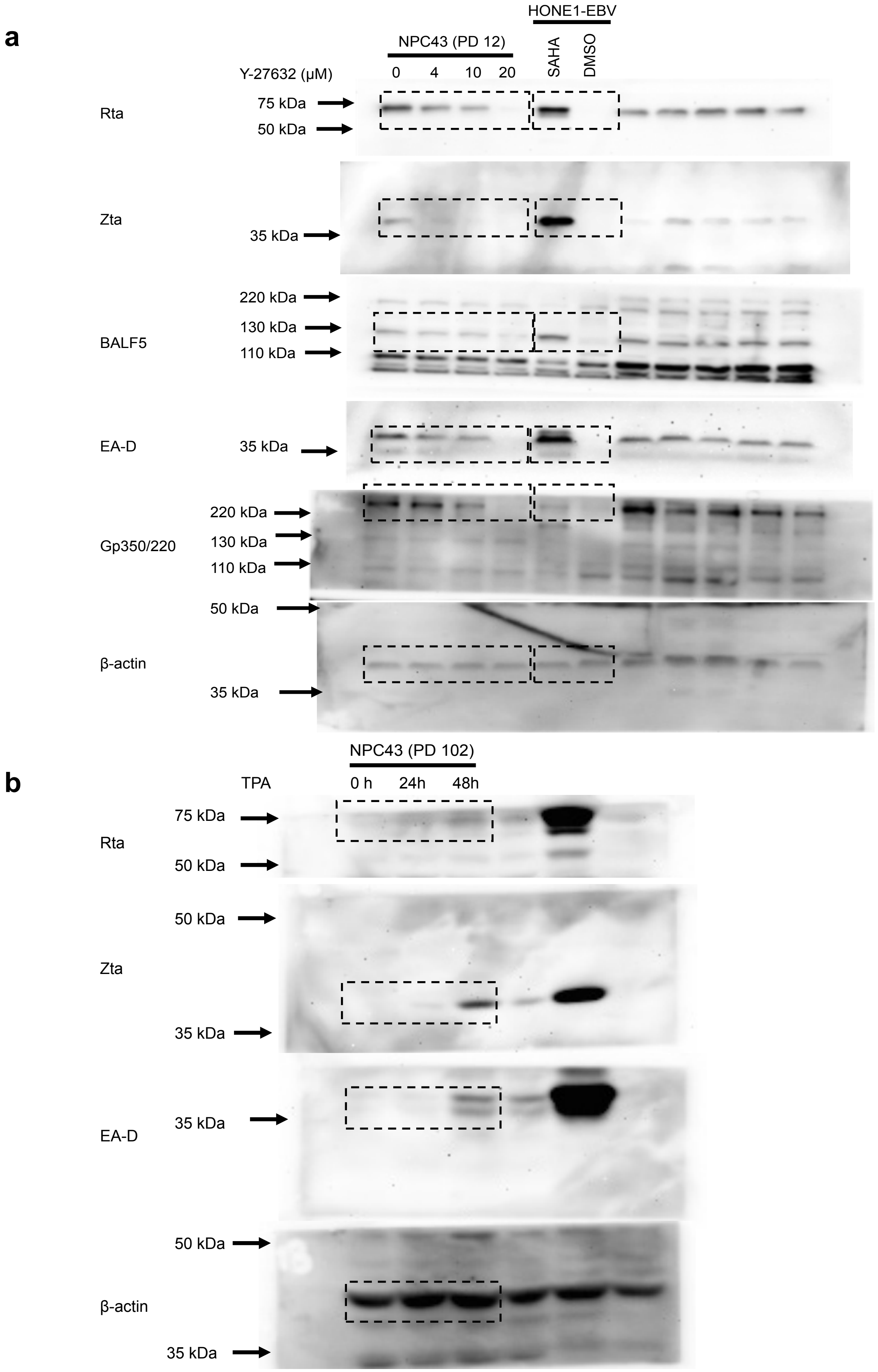

Supplementary Figure 25. Uncropped images of Western blot membranes.
a) Images corresponding to experiments shown in Figure 4c.
b) Images corresponding to experiments shown in Figure 5a.

Supplementary Tables

Supplementary Table 1. STR profiles.

	AMEL	*D5S818*	*D13S317*	*D7S820*	*D16S539*	*vWA*	*TH01*	*TPOX*	*CSF1PO*	*D3S1358*	*D21S11*	*D18S51*	*Penta E*	*Penta D*	*D8S1179*	*FGA*	*D19S433*	*D2S1338*
Patient 23	X, Y	11, 12	8, 13	11, 12	10, 11	19	9, 10	8, 11	13	16, 17	30, 33.2	15, 16	NA[*]	NA	10, 14	22, 24	13, 14	19, 24
Xeno23	X, Y	11, 12	8, 13	11, 12	10	19	9, 10	8, 11	13	17	33.2	15, 16	NA	NA	10, 14	22, 24	13, 14	19, 24
Patient 32	X	10, 13	8, 12	10, 11	9, 10	16, 18	7	8, 10	10, 11	15, 16	29, 31	14, 15	NA	NA	11, 12	24	13, 14.2	19, 20
Xeno32	X	10	8	10, 11	9, 10	16, 18	7	8, 10	10, 11	15	29, 31	14, 15	NA	NA	11, 12	24	13, 14.2	19, 20
Patient 47	X	12, 13	9, 10	10, 12	10, 11	14, 16	7, 10	8, 9	12	16, 17	30, 32.2	13	11, 19	14	15	16, 24	NA	NA
Xeno47	X	12	9, 10	10, 12	11	14, 16	7, 10	8, 9	12	17	30, 32.2	13	11, 19	14	15	16, 24	NA	NA
Patient 76	X	11, 13	10, 11	9, 11	10, 11	14, 17	7, 9	8, 12	12, 13	16, 17	30, 32.2	13, 15	5, 13	7, 9	10, 11	19, 25	NA	NA
Xeno76	X	11, 13	10, 11	9, 11	11	14, 17	7, 9	8, 12	12, 13	17	32.2	13, 15	5, 13	9	10, 11	19, 25	NA	NA
X2117	X, Y	12	10	11	11, 12	15, 17, 18	7	8, 12	12	15	29, 30	16, 19	NA	NA	14, 16	22	13, 14	19, 20
C15	X	10, 13	11, 14	11, 12	11, 12	16, 17	9	8, 11	11, 16	17, 18	27, 30	17, 18	NA	NA	13, 14	20, 22	14.2, 16.2	16, 24
C17	X, Y	11, 14	11	11	12	14, 15	6	9	16	15	30	20	NA	NA	11, 14, 15	23	15, 16	19, 20
C666-1	X, Y	11, 12	8, 11	11, 12	10	17, 18	6, 8	8, 11	11, 15, 16	16, 17	28, 29, 30.2, 31.2	16	NA	NA	11, 13, 14, 15	23, 24	13, 15.2	16, 23
Patient 43	X, Y	11, 13	10, 12	12, 13	10	14, 16	7, 9	8	10, 13	15, 18	28, 32	13, 15	11, 13	8, 13	11	18, 22	NA	NA
NPC43 (PD 2)	X	11	12	12, 13	10	14, 16	7	8	13	18	32	13, 15	ND[†]	8	11	22	NA	NA
NPC43 (PD 17)	X	11	12	12, 13	10	14, 16	7	8	13	18	32	13, 15	ND	8	11	22	NA	NA
NPC43 (PD 108)	X	11	12	12, 13	10	14, 16	7	8	13	18	32	13, 15	ND	8	11	22	NA	NA
Patient 38	X	10, 13	8, 9	8, 11	10, 12	17, 18	6, 9	8, 11	10, 11	16, 18	29, 33.2	13, 21	NA	NA	12, 13	24, 26	11, 14.2	23, 24
NPC38	X	10, 12	8, 9, 10	8, 10, 11, 12	10	18	6, 9	8 10	12, 13	18, 19	30, 33.2	13, 21	NA	NA	12, 13, 14	23, 25	13.2 15.2	23, 25
Patient 53	X, Y	11, 12	9, 11	9.1, 11	11	14, 17	7, 9	8, 9	11, 13	15	28, 29	13, 15	15, 16	9, 11	14, 15	24	NA	NA
NPC53	X	11, 12	9, 11	9.1, 11	11	14, 17	9	8, 9	11, 13	15	28	13, 15	15, 16	11	14, 15	24	NA	NA

[*]NA, not available. [†]ND, not detectable.

Supplementary Table 2. Clinical information of cases which failed to establish PDX or cell line.

Case No.	Clinical status				Sample type	Tumor recurrence	Tumor metastasis
	T	N	M	Overall Staging			
20*	1	2	0	NA†	Nasopharyngectomized tissue	Yes	No
21*	3	2	0	III	Nasopharyngectomized tissue	Yes	Yes
22*	2	1	0	II	Nasopharyngectomized tissue	Yes	Yes
24	3	2	0	III	Nasopharyngectomized tissue	Yes	No
25	2	0	0	II	Nasopharyngectomized tissue	Yes	No
27*	1	1	0	II	Nasopharyngectomized tissue	Yes	No
36	2	1	0	IIB	Primary biopsy	No	No
37	2	0	0	II	Nasopharyngectomized tissue	Yes	No
39	3	1	0	III	Nasopharyngectomized tissue	Yes	No
42	3	2	0	III	Primary biopsy	No	No
51	3	2	0	III	Primary biopsy	No	No
52*	3	1	0	III	Nasopharyngectomized tissue	Yes	No
55	3	3b	0	IVB	Primary biopsy	No	No
57	3	1	0	III	Primary biopsy	No	No
58	1	1	0	IIB	Primary biopsy	No	No
59*	NA	NA	NA	IVC	Primary biopsy	No	Yes
60	2	3b	0	IVB	Primary biopsy	Yes	Yes
61	1	2	0	III	Primary biopsy	No	No

*Patients who did not survive by June 30, 2018.
†NA, not available.

Supplementary Table 3. Tumor content of patient tissues used for transplantation and patient plasma EBV copy number.

Patient No.	Tumor content	Plasma EBV copy number (copies/ml)
20	45%	NA[†]
21	45%	NA
22	40%	187083
23[*]	80%	740
24	20%	0
25	75%	97
27	0%	421
32[*]	NA	845
36	0%	515
37	0%	37
38[*]	NA	0
39	0%	348
42	10%	1717
43[*]	5%	38
47[*]	<5%	2646
51	10%	549
52	9%	337
53[*]	33%	4171
55	50%	0
57	5%	1392
58	40%	4446
59	0%	4254
60	0%	2642
61	5%	15
76[*]	NA	0

[*]Successful establishment of PDXs or cell lines from patient tumor tissues.
[†]NA, not available.

Supplementary Table 4. Sequencing reads filtering and mapping.

Sample	No. of raw reads	No. of filtered reads	Percentage of filtered reads (%)	No. of reads mapped to hg38	Percentage of reads mapped to hg38 (%)
HK1	131,298,628	110,637,516	84.26	106,830,138	96.56
NPC38	120,648,234	108,746,656	90.14	104,798,210	96.37
NPC53	111,695,622	109,675,566	98.19	105,625,286	96.31
NPC43	119,761,832	106,569,038	88.98	102,659,506	96.33
C17	124,300,026	110,340,226	88.77	105,705,308	95.80
C666-1	119,531,820	107,589,958	90.01	103,236,314	95.95
Xeno23	117,931,204	114,657,166	97.22	107,010,490	93.33
Xeno32	114,405,752	105,282,044	92.03	92,819,780	88.16
Xeno47	116,307,818	103,466,154	88.96	96,941,386	93.69
Xeno76	119,752,488	112,685,428	94.10	104,119,398	92.40

Supplementary Table 5. Mapping information of non-human and non-mouse reads from WGS to reference EBV genome in newly established xenografts and cell lines.

Sample	Total Reads*	Reads mapped to NC_007605	% of Reads mapped to EBV
Xeno23	30,368,832	5,334,489	17.57
Xeno32	28,375,740	2,735,353	9.64
Xeno47	44,694,994	4,111,462	9.2
Xeno76	43,039,072	4,378,253	10.17
NPC43	32,711,200	2,195,343	6.71
NPC38	36,159,780	0	0

*Total Reads: reads mapped to human and mouse from WGS were removed, and the residue reads were counted.

Supplementary Table 6. Information of EBV *de novo* assembly.

Sample	Max contig	N50	k-mer length	Depth
Xeno23	44,051	19,912	35	3800 - 4200
Xeno32	41,789	28,075	35	300 - 500
Xeno47	44,084	29,802	35	2800 - 3200
Xeno76	44,055	19,797	35	4000 - 4400
NPC43	44,063	19,803	35	1600 -2000

Supplementary Table 7. Primers and probes for real-time PCR.

Gene	Probe	Primer sequence (5' to 3')
β-globin	17	Forward: GGCCCTTTTGCTAATCATGT
		Reverse: CACACAGACCAGCACGTTG
EBNA1	16	Forward: GAGAAGGCCCAAGCACTG
		Reverse: CTCCTTGACCACGATGCTTT
EBER1/2	5	Forward: GAGGTTTTGCTAGGGAGGAGA
		Reverse: CACCACCCGGGACTTGTA
LMP1	10	Forward: GTCCTGTGGGCCATTGTC
		Reverse: CCCACTCTGCTCTCAAAACC
BZLF1	48	Forward: CCGGCTTGGTTAGTCTGTTG
		Reverse: AGCTTATGCAGCACCTCAGC
BRLF1	38	Forward: GCTCAGGTCCATCTGTCCAC
		Reverse: GGGAGATGGCTGACACTGTT
BMRF1	29	Forward: GCGAGGAAAAGGACATCGT
		Reverse: CTTCACTTTCTTGGGGTGCT
BLLF1	55	Forward: CATCTACAGATTCCAGGCTTACTTG
		Reverse: AGCTTCCAATTAACGTCACCA
GAPDH	60	Forward: AGCCACATCGCTCAGACAC
		Reverse: GCCCAATACGACCAAATCC
