# Supplementary material for: Establishment and characterization of new tumor xenografts and cancer cell lines from EBV-positive nasopharyngeal carcinoma
Source: Nat Commun. 2018 Nov 7;9:4663. doi: 10.1038/s41467-018-06889-5 (PMC6220246; doi:10.1038/s41467-018-06889-5)
Supplement: Supplementary file 6 — Description of Additional Supplementary Files [file 41467_2018_6889_MOESM6_ESM.docx]

**Title:** Supplementary Data 1.
**Description:** Nonsynomous SNVs and indels identified in NPC cell line and xenografts

**Title:** Supplementary Data 2.
**Description:** Structural variants identified in NPC cell line and xenografts

**Title:** Supplementary Data 3.
**Description:** Homozygous deletion and amplification regions identified in NPC cell lines and xenografts.
